# Implementation of guideline-recommended organ-protective therapy in people with type 2 diabetes and cardiovascular disease, heart failure, or kidney disease: real-world evidence from a German University Hospital

**DOI:** 10.3389/fendo.2025.1736984

**Published:** 2026-01-06

**Authors:** Karzan Suliman, Young Hee Lee-Barkey, Alexander Brehmer, Bernd Stratmann, Jasmin M. Klose, Tim Lenfers, Jens Kleesiek, Susanne Reger-Tan, Julius Keyl

**Affiliations:** 1 Department of Diabetes and Endocrinology, Heart and Diabetes Center NRW, University Hospital of Ruhr-University Bochum, Bochum, Germany; 2 Institute for Artificial Intelligence in Medicine (IKIM), University Hospital Essen, Essen, Germany; 3 Institute of Pathology, University Hospital Essen, Essen, Germany

**Keywords:** atherosclerotic cardiovascular disease, chronic kidney disease, GLP1RA, guidelines, heart failure, organ protection, SGLT2i, type 2 diabetes

## Abstract

**Background:**

Organ-protective therapy with sodium-glucose cotransporter 2 inhibitors (SGLT2i) and glucagon-like peptide-1 receptor agonists (GLP1RA) is recommended for people with type 2 diabetes (PwT2D) and cardiorenal comorbidities, yet real-world uptake remains uncertain.

**Methods:**

Using the University Hospital Essen FHIR data lake (2019–2024), we identified PwT2D and cardiorenal comorbidities qualifying for organ-protective therapy [atherosclerotic cardiovascular disease (ASCVD/high ASCVD risk), heart failure (HF), chronic kidney disease (CKD)] by International Classification of Diseases (ICD-10) diagnoses, German surgery procedure codes (OPS), and clinical data. Guideline adherence and treatment trends were assessed by Anatomical Therapeutic Chemical (ATC) medication data for SGLT2i and GLP1RA. Group differences and factors associated with guideline adherent therapy were analyzed using X²-test and multivariable modeling.

**Results:**

The total cohort comprised 19,684 individuals (age: 70.3 ± 11.2 years, female sex: 36.8%, mean HbA1c 7.2 ± 1.5%, BMI 29.7 ± 6.4 kg/m²). In group analysis, female sex was the most prominent difference with lower rates of women among those treated with SGLT2i and/or GLP1RA with small to moderate effect size (standardized mean difference 0.48). Guideline adherent therapy with SGLT2i and/GLP1RA increased steadily from 10.2% to 48.7% in PwT2D eligible for organ-protective therapy. Treatment rates varied by comorbidity and drug class, with the lowest overall uptake observed in people with T2D and CKD (32.5%) and the highest in those with all three cardiorenal comorbidities (63.9%). Among drug classes, SGLT2i use was 44.7%, GLP1RA use was 9.5%, and combination therapy remained low at 5.5%. Female sex (OR 0.76, 95% CI 0.66-0.89, p<0.001), and higher age (OR 0.98, 95% CI 0.99-0.97, p<0.0001) reduced the likelihood, while higher number of comorbidities (OR 1.26, 95% CI 1.18-1.34, p<0.0001) and higher number of medication (OR 1.98, 95% CI 1.88-2.08, p<0.0001) increased the likelihood of guideline adherent therapy.

**Conclusions:**

Guideline recommendations for organ protection in PwT2D and cardiorenal comorbidities are increasingly reflected in clinical practice, yet a substantial care gap persists, with the majority of individuals remaining untreated. Certain subpopulations - particularly women - are underrepresented among those receiving guideline adherent therapy. Further research into the causes of undertreatment and development of targeted implementation strategies is needed to close remaining evidence–practice gaps.

## 1 Introduction

Driven by robust outcome trials demonstrating that sodium–glucose cotransporter 2 inhibitors (SGLT2i) and glucagon-like peptide 1 receptor agonists (GLP1RA) reduce major adverse cardiovascular events, heart failure hospitalizations, and progression of kidney disease, international and national guidelines recommend prioritizing pharmacologic treatment with proven cardiovascular and renal benefits in people with type 2 diabetes (PwT2D) evolving towards organ protection as primary therapeutic target (1–4). In 2019, the American Diabetes Association (ADA) and the European Association for the Study of Diabetes (EASD) have recommended the use of SGLT2i and GLP1RA in PwT2D and established atherosclerotic cardiovascular disease (ASCVD), heart failure (HF), and/or chronic kidney disease (CKD) emphasizing that SGLT2i or GLP1RA should be initiated in those individuals at high risk for cardiovascular and renal events (5). This guideline initiated a transformative paradigm shift from glucose-centric diabetes management towards a comprehensive, organ-protective strategy. Among PwT2D, eligibility for organ-protective therapy is high with approximately 50% according to ADA/EASD and 80% according to ESC guidelines (6). However, electronic health record and national registry data show that real-world implementation of these recommendations remains low (6–9). Special populations such as Black and African Americans, women, elderly, and patients hospitalized in the year prior or without secondary preventive medication are described to be at higher risk to not receive guideline-guided treatment (8, 9). In Germany, evidence remains scarce, and the underlying barriers are yet to be elucidated. This study aims to quantify real-world trends in SGLT2i and GLP1RA use among PwT2D and cardiorenal comorbidity and to identify factors associated with non-guideline adherent therapy.

## 2 Materials and methods

### 2.1 Data source and infrastructure

Data were extracted from the University Hospital Essen Clinical Data Lake, a large-scale federated data environment built on the Fast Healthcare Interoperability Resources (FHIR) standard. The system integrates structured and semi-structured data from the hospital’s electronic health record (EHR), including diagnoses, medication, laboratory results, and clinical documentation. This architecture follows the approach described by Brehmer et al. (10), who developed a FHIR-based data integration and analytics platform enabling semantic interoperability and large-scale real-world evidence generation. By 2019 to 2024, the data lake currently contains over two billion FHIR resources, encompassing inpatient and outpatient encounters, laboratory information systems, medication orders, and billing data. All analyses were conducted within a secure research environment using the open-source Python client FHIRPACK and the FHIR Analytics Network (FHAN) toolkit, which enable standardized cohort definition, resource querying, and data harmonization in compliance with FAIR (Findable, Accessible, Interoperable, Reusable) principles.

International Classification of Diseases, Tenth Revision (ICD-10) diagnoses, German surgery procedure codes (OPS), Anatomical Therapeutic Chemical (ATC) medication codes, and clinical data from electronic health records (EHR) were used as data source information for the analysis. ICD-10 code E11 was used to identify people with type 2 diabetes (PwT2D). Cardiorenal comorbidities were stratified by ICD-10 codes, OPS procedures and clinical data extracted from the EHR used to identify those patients with indication for organ-protective treatment with SGLT2i and/or GLP1RA. (**Supplementary Data 1.1**).

### 2.2 Cohort definition

Cases fulfilling the following in- and exclusion criteria were extracted from the University Hospital Essen Clinical Data Lake to analyze PwT2D and at least one cardiorenal disease qualifying for guideline adherent therapy with SGLT2i and/or GLP1RA during the observation period 2019-2024. As the first guideline recommendations for the use of SGLT2i/GLP1RA for organ protection were published by the ADA/EASD in 2019, this year was chosen as starting point of the observation period (5). Established ASCVD/high ASCVD risk, HF and CKD were regarded as cardiorenal comorbidity qualifying for organ-protective treatment with SGLT2i and/or GLP1RA.

#### 2.2.1 Inclusion criteria

- Adults (age ≥ 18 years)
- Diagnosis of T2D (E11)
- Presence of at least one of the following conditions defining cardiorenal comorbidity:

1a. Established ASCVD defined by ICD-10 or OPS code for coronary artery disease, stroke or atherosclerosis including peripheral artery disease (I20, I21, I25, I63, I70, 5-363.4, 8-836, 8-837; 8-849, 8-981)

1b. High ASCVD risk defined by age >55 years and presence of at least two cardiovascular risk factors such as hypertension (I10, I15), obesity (E66) and/or BMI >30 kg/m², dyslipidemia (E78), albuminuria (R80) and/or smoking).

2. Heart failure (HF; I50) and/or

3. Chronic kidney disease (CKD; N18, R80).

#### 2.2.2 Exclusion criteria

- Missing entry of medication data
- Implausible entries (e.g. age < 18 years)

**Figure 1:**
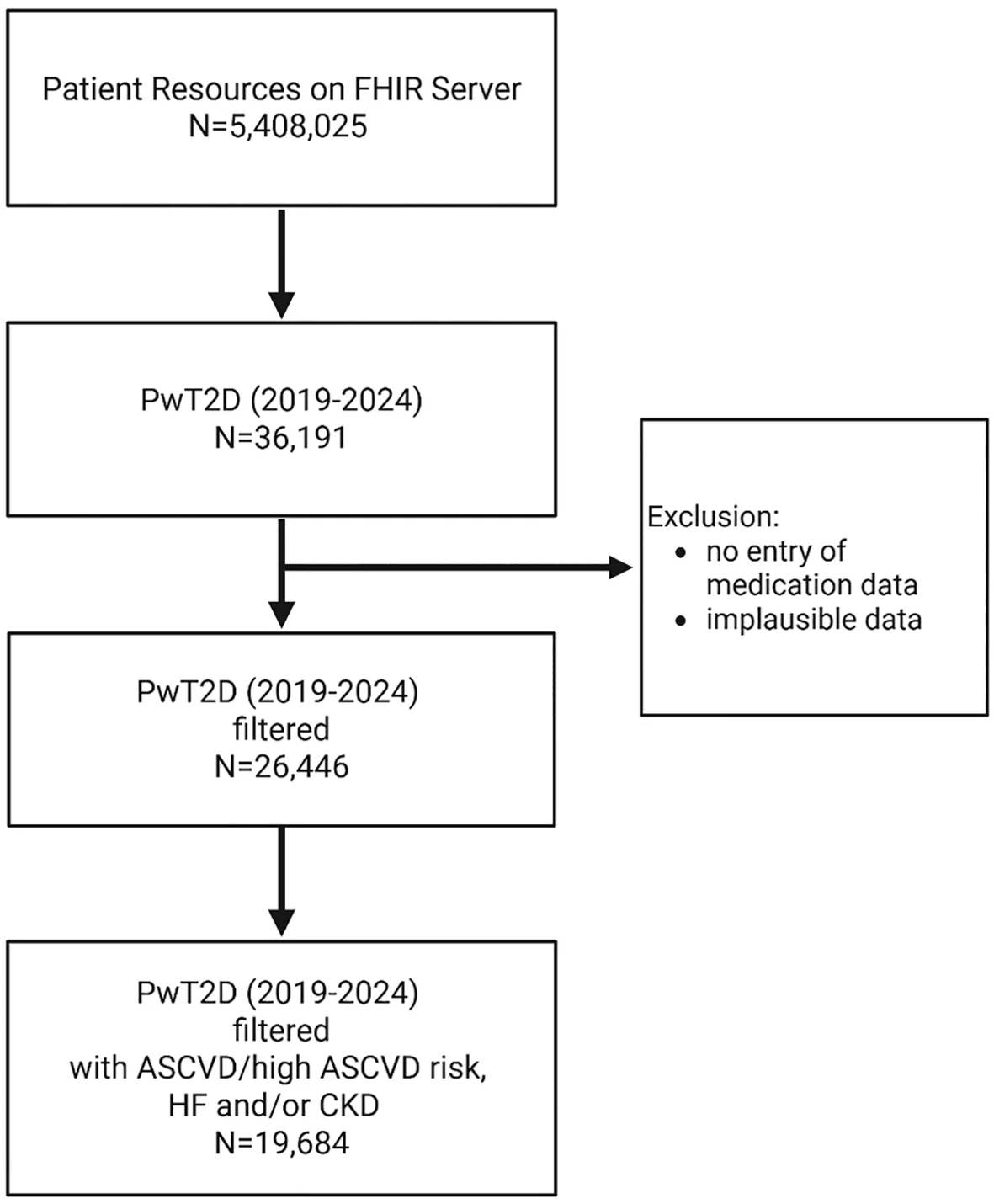
Flowchart of study cohort selection. Illustration of the selection process for the final total cohort. From an initial pool of resources available on the FHIR server (N = 5,408,025), 36,191 PwT2D were identified. After excluding PwT2D without medication data and/or with implausible values, 26,446 PwT2D remained. Among these, 19,684 with documented ASCVD/high ASCVD risk, HF and/or CKD were identified. PwT2D, people with type 2 diabetes; ASCVD, atherosclerotic cardiovascular disease; HF, heart failure; CKD, chronic kidney disease. Created in https://BioRender.com.

Following the flow of this study cohort selection, the final total cohort comprises PwT2D and at least one cardiorenal comorbidity (ASCVD/indicators of high CVD risk, HF and/or CKD) (**Figure 1**).

Comorbidity count summarizes the number of the following five cardiorenal diseases to reflect cardiorenal multimorbidity: 1) coronary artery disease defined by one of the following I20, I21, I25, OPS 5-363.4, 8-837, 2) stroke defined by one of the following I63, OPS 8-981, 3) atherosclerosis defined by one of the following I70, OPS 8-836, 8-849, 4) heart failure defined by I50 and 5) CKD defined by one of the following N18, R80.

### 2.3 Medication and treatment identification

Using ATC codes, medication history was extracted from a FHIR-based server using specific queries on the “MedicationStatement” (data source: patient history), “MedicationRequest” (data source: medication order), and “MedicationAdministration” (data source: medication application). The date of the first administration per class was used to compute temporal trends in medication uptake and medication count: 1) ‘Metformin’, 2) ‘Lipidsenker’ (lipidlowering drugs), 3) ‘DPP-4’ (dipeptidyl peptidase inhibitors), 4) ‘SGLT’,’ (SGLT2i), 5) ‘Insulin’, 6) ‘Other A10B Code’, 7) ‘GLP’ (GLP1RA), 8) ‘Sulfonylharnstoffe’ (sulfonylurea), 9) ‘Glinide’ (glinides), 10) ‘Antihypertensiva_C03’, 11) ‘Antihypertensiva_C09’, 12) ‘Antihypertensiva_C08’, 13) ‘Antihypertensiva_C07’, 14) ‘Antihypertensiva_C02’ (blood pressure lowering drugs), 15) ‘Acarbose’.

Guideline adherent treatment was defined as documented medication history of SGLT2i and/or GLP1RA in PwT2D and cardiorenal comorbidity (5).

### 2.4 Clinical and demographic variables

Demographic and clinical variables were retrieved from EHR and laboratory FHIR resources, including age, body mass index (BMI), sex, glycosylated hemoglobin (HbA1c), total cholesterol, high density lipoprotein (HDL) cholesterol, low density lipoprotein (LDL) cholesterol, triglycerides, aspartate-aminotransferase (ASAT), alanine-aminotransferase (ALAT), N-terminal pro-brain natriuretic peptide (NT-proBNP), and estimated glomerular filtration rate (eGFR).

### 2.5 Temporal trends and treatment uptake

Annual rates for medication with SGLT2i and GLP1RA were evaluated in all PwT2D and at least one cardiorenal comorbidity during the observation period 2019 – 2024. Temporal changes were analyzed year by year using the Х^2^-test for trend.

### 2.6 Multivariable modeling of guideline adherent therapy

The dependent variable was defined as being treated with SGLT2i and/or GLP1RA. Independent variables included age, BMI, female sex, HbA1c, eGFR, number of comorbidities (comorbidity count), and number of drugs (medication count). Multivariate logistic regression was applied to identify factors associated with guideline adherent treatment. Adjusted odds ratios (OR) with 95% confidence intervals (CI) were reported. Model diagnostics included the Hosmer-Lemeshow test and variance inflation factors to assess model fit and multicollinearity. Analysis of continuous variables was performed by unpaired t-test and analysis of categorical variables by Х^2^-test following Fisher’s exact test comparing non-guideline adherent therapy to guideline adherent therapy. OR were analyzed by using Baptista-Pike method or calculated as Gart adjusted logit interval, respectively. Analysis for linear trend over time was performed by applying Х^2^-test for trend.

### 2.7 Data quality, ethics, and governance

Data extraction followed standardized FHIR queries via the python package FHAN (https://github.com/trostalski/fhan). Quality control included plausibility checks (e.g., age < 18 years), removal of duplicates, and consistency validation across resource types. All data were pseudonymized prior to analysis. The study was conducted in accordance with the Declaration of Helsinki and approved by the Ethics Committee of the University of Duisburg-Essen (Reference 22-10881-BO). The study design complies with German data protection law (DSGVO/GDPR). No informed consent was required as routinely collected health care data were used.

### 2.8 Statistical environment

All statistical analyses were performed using Python 3.13 (libraries: pandas, numpy, statsmodels, scipy) and GraphPad Prism 10.1.2 (324). Visualizations were created using matplotlib and seaborn or biorender.

The approach to extracting diagnostic information via FHIR Condition and Procedure resources wasalready demonstrated to reliably map EHR entities into interoperable clinical phenotypes within a hospital-wide data lake. FHIRPACK and FHAN enabled harmonization of units, transformation of value sets, and normalization across different source systems - mirroring a multi-modal data integration strategy. The analytic workflow and FHIR-based cohort construction followed the principles of longitudinal data linkage, data extraction and semantic harmonization and were conducted via FHIRPACK v2.0 and the FHAN analytics environment, following the framework established by Brehmer et al. (10).

## 3 Results

### 3.1 Study population

To identify eligible patients, information on ICD-10 codes and OPS procedures was used from the University Hospital Essen FHIR data lake which comprises 5,408,025 FHIR Patient Resources (**Figure 1**). In the observation period 2019 to 2024, 36,191 cases were classified as PwT2D according to ICD-10 code E11. Exclusion of individuals without medication data and/or with implausible data condensed the cohort to 26,446 PwT2D. After application of ICD-10 and OPS-procedures and clinical data defining cardiorenal comorbidities (ASCVD/indicators of high CVD risk, and/or HF, and/or CKD), a final cohort of 19,684 PwT2D with at least one cardiorenal comorbidity classifying for guideline adherent treatment with SGLT2i and/or GLP1RA was identified. This patient cohort had a mean HbA1c of 7.2 ± 1.5% (54.6 ± 16.4 mmol/mol), BMI of 29.7 ± 6.4 kg/m² and an LDL-cholesterol above target (102.6 ± 46.1 mg/dl). The most common diagnoses were hypertension (82.7%), dyslipidemia (59.5%) and coronary artery disease (57.5%). Insulin (41.6%), metformin (40.1%) and SGLT2i (30.2%) were the most commonly used diabetes medications. (**Table 1**)

**Table 1:** Characteristics of the PwT2D and cardiorenal comorbidity according to guideline adherent therapy (2019–2024).

Parameter	Total Cohort of PwT2D and cardiorenal comorbidity (2019-2024)	Treated with SGLT2i and/or GLP1RA	Not treated with SGLT2i and/or GLP1RA	P for difference between subgroups	SMD
Demographics					
Number (N)	19,684	6,730	12,954		
Age (years)	70.3 ± 11.2	69.1 ± 11.0	70.9 ± 11.2	p<0.0001	0.16
BMI (kg/m²)	29.7 ± 6.4	30.1 ± 6.4	29.5 ± 6.3	p<0.0001	0.10
Female sex (%)	7,238 (36.8)	2,131 (31.7)	5,107 (39.4)	p<0.0001	0.48
Systolic blood pressure (mmHg)	123.1 ± 37.2	120.2 ± 35.6	124.6 ± 37.9	p<0.0001	0.12
Diastolic blood pressure (mmHg)	67.5 ± 20.9	67.0 ± 20.1	67.7 ± 21.2	p=0.017	0.03
**Laboratory values**	**Mean ± SD**	**Mean ± SD**	**Mean ± SD**		
HbA1c (%); HbA1c (mmol/mol)	7.2 ± 1.5; 54.6 ± 16.4	7.4 ± 1.6; 56.9 ± 17.6	7.0 ± 1.4; 53.2 ± 15.4	p<0.0001	0.23
Total cholesterol (mg/dl)	156.8 ± 53.0	151.9 ± 53.4	159.7 ± 52.6	p<0.0001	0.15
LDL cholesterol (mg/dl)	102.6 ± 46.1	98.8 ± 45.7	104.8 ± 46.1	p<0.0001	0.13
HDL cholesterol (mg/dl)	42.8 ± 14.6	41.5 ± 13.7	43.5 ± 15.0	p<0.0001	0.17
Triglycerides (mg/dl)	175.6 ± 151.0	181.1 ± 190.0	172.4 ± 111.9	p=0.014	0.06
ASAT (U/l)	58.9 ± 574.0	49.6 ± 407.9	63.8 ± 643.9	p=ns	0.02
ALAT (U/l)	40.8 ± 157.3	41.1 ± 144.3	40.7 ± 163.7	p=ns	0.002
NT-proBNP	4,456 ± 11,739	3,795 ± 9,473	4,956 ± 13,178	p<0.0001	0.09
eGFR (mL/min/1.73m²)	53.5 ± 14.3	55.0 ± 11.8	52.7 ± 15.5	p<0.0001	0.16
**Comorbidities**	**N (%)**	**N (%)**	**N (%)**		
Coronary artery disease	11,326 (57.5)	4,497 (66.8)	6,829 (52.7)	p<0.0001	0.29
Stroke	1,813 (9.2)	519 (7.7)	1,294 (10.0)	p<0.0001	0.08
Peripheral artery disease	4,144 (21.1)	1,740 (25.9)	2,404 (18.6)	p<0.0001	0.18
Obesity	9,768 (49.6)	3,590 (53.3)	6,178 (47.7)	p<0.0001	0.11
Hypertension	16,285 (82.7)	5,543 (82.4)	10,742 (82.9)	p=ns	0.01
Dyslipidemia	11,704 (59.5)	4,639 (68.9)	7,065 (54.5)	p<0.0001	0.29
Heart failure	9,298 (47.2)	4,025 (59.8)	5,273 (40.7)	p<0.0001	0.38
Chronic kidney disease	6,296 (32.0)	2,127 (31.6)	4,169 (32.2)	p=ns	0.01
Current smoker	966 (4.9)	297 (4.4)	669 (5.2)	p<0.05	0.03
Medication	N (%)	N (%)	N (%)		
Insulin	8,194 (41,6)	3,129 (46.5)	5,065 (39.1)	p<0.0001	0.14
Biguanides	7,894 (40.1)	3,257 (48.4)	4,637 (35.8)	p<0.0001	0.26
SGLT2i	5,936 (30.2)	5,936 (88.2)	0	p<0.0001	NA
DPP4i	3,228 (16.4)	1,048 (15.6)	2,180 (16.8)	p<0.0001	0.03
GLP1RA	1,574 (7.6)	1,574 /23.4)	0	p<0.0001	NA
Sulfonylureas	413 (2.1)	168 (2.5)	245 (2)	p<0.01	0.04
Glinides	190 (1.0)	87 (1.3)	103 (0.8)	p<0.001	0.05
Other OAD	1139 (5.8%)	487 (7.2)	652 (5.0)	p<0.0001	0.09
ACEi/ARBs	13,505 (68.6)	5,256 (78.1)	8,249 (63.7)	p<0.0001	0.31
Beta blocker	12,638 (64.2)	4,957 (73.7)	7,681 (59.3)	p<0.0001	0.30
Diuretics	10,336 (53.5)	4,333 (64.3)	6,003 (46.3)	p<0.0001	0.36
Calcium channel blocker	6,806 (34.6)	2,317 (34.4)	4,489 (34.7)	p=ns	0.005
Other hypertensive drugs	2,371 (12.1)	787 (11.7)	1,584 (12.2)	p=ns	0.02
Lipid lowering medication	13,600 (69.1)	5,471(81.3)	8,129 (62.8)	p<0.0001	0.40

Demographic, clinical, biochemical characteristics and medication data of the study population are presented with continuous variables presented as mean ± standard deviation (SD) and categorical variables as absolute numbers (N) and percentage affected (%). Analysis of continuous variables by unpaired t-test, analysis of categorical variables by Х^2^-test following Fisher’s exact test comparing PwT2D and cardiorenal comorbidity treated with SGLT2i and/or GLP1RA vs. not treated with SGLT2i and/or GLP1RA.

ACEi/ARB, angiotensin-converting enzyme inhibitor/angiotensin receptor blocker; ALAT, alanine-aminotransferase; ASAT, aspartate-aminotransferase; BMI, body mass index; DPP4i, dipeptidyl peptidase 4-inhibitor; eGFR, estimated glomerular filtration rate; GLP1RA, glucagon-like peptide 1 receptor agonist; HDL-cholesterol, high density lipoprotein-cholesterol; LDL-cholesterol, low density lipoprotein-cholesterol; NA, not applicable; NT-proBNP, N-terminal pro-brain natriuretic peptide; OAD, oral antidiabetics; SGLT2i, Sodium–glucose cotransporter 2 inhibitor; SMD, standardized mean difference; NA, not applicable.

In the total cohort of 19,684 PwT2D and cardiorenal comorbidity, 6,730 (34.2%) received guideline adherent therapy with SGLT2i and/or GLP1RA during the observation period. Several demographic and clinical characteristics differed significantly between these two groups, with standardized mean differences (SMD) indicating the magnitude of imbalance beyond statistical significance of minimal to moderate size. PwT1D receiving guideline adherent therapy were generally younger and less often female, with small-to-moderate SMD (0.16 for age and 0.48 for sex), the latter indicating a substantial imbalance. They also showed higher BMI, lower and blood pressure values, and blood pressure values with only small differences between groups (SMD ≤0.12). A moderate imbalance in HbA1c (SMD 0.23) was observed, with guideline adherent treated patients exhibiting slightly higher glycemic levels. Lipid levels, consistently showed small imbalances (SMD 0.13–0.17), with more favorable lipid profiles observed in the guideline adherent group. Kidney function was modestly better in patients receiving guideline therapy, reflected by an eGFR SMD of 0.16. Comorbidity patterns displayed some of the strongest imbalances. Coronary artery disease, dyslipidemia, and particularly heart failure were considerably more prevalent in the guideline-adherent group, with SMDs of 0.29-0.38, respectively. Conditions such as hypertension and chronic kidney disease, however, showed negligible differences (SMD ≤0.01). Medication use demonstrated expected differences, given that some agents define guideline adherence. The use of SGLT2i and GLP1RA was exclusive to the guideline adherent subgroup. Beyond these, substantial imbalances were observed in the use of ACE inhibitors/ARB, beta-blockers, diuretics, and lipid-lowering therapies (SMD 0.30–0.40), all more frequently prescribed to patients receiving guideline-based treatment. Other glucose-lowering agents showed only small or negligible differences. Use of other glucose-lowering agents varied: insulin and metformin were more frequently prescribed in the guideline adherent treated subgroup, while DPP4i, sulfonylureas, and glinides were slightly less common. Consistent with higher cardiovascular risk, treated individuals more often received ACE inhibitors/ARBs, beta-blockers, diuretics, and lipid-lowering therapies. Overall, patients receiving guideline adherent therapy differed in small to moderate extent from those who did not, particularly with respect to cardiovascular comorbidities, renal function, glycemic control, and cardiometabolic medication use.

### 3.2 Medication trends for SGLT2i and GLP1RA

**Figure 2:**
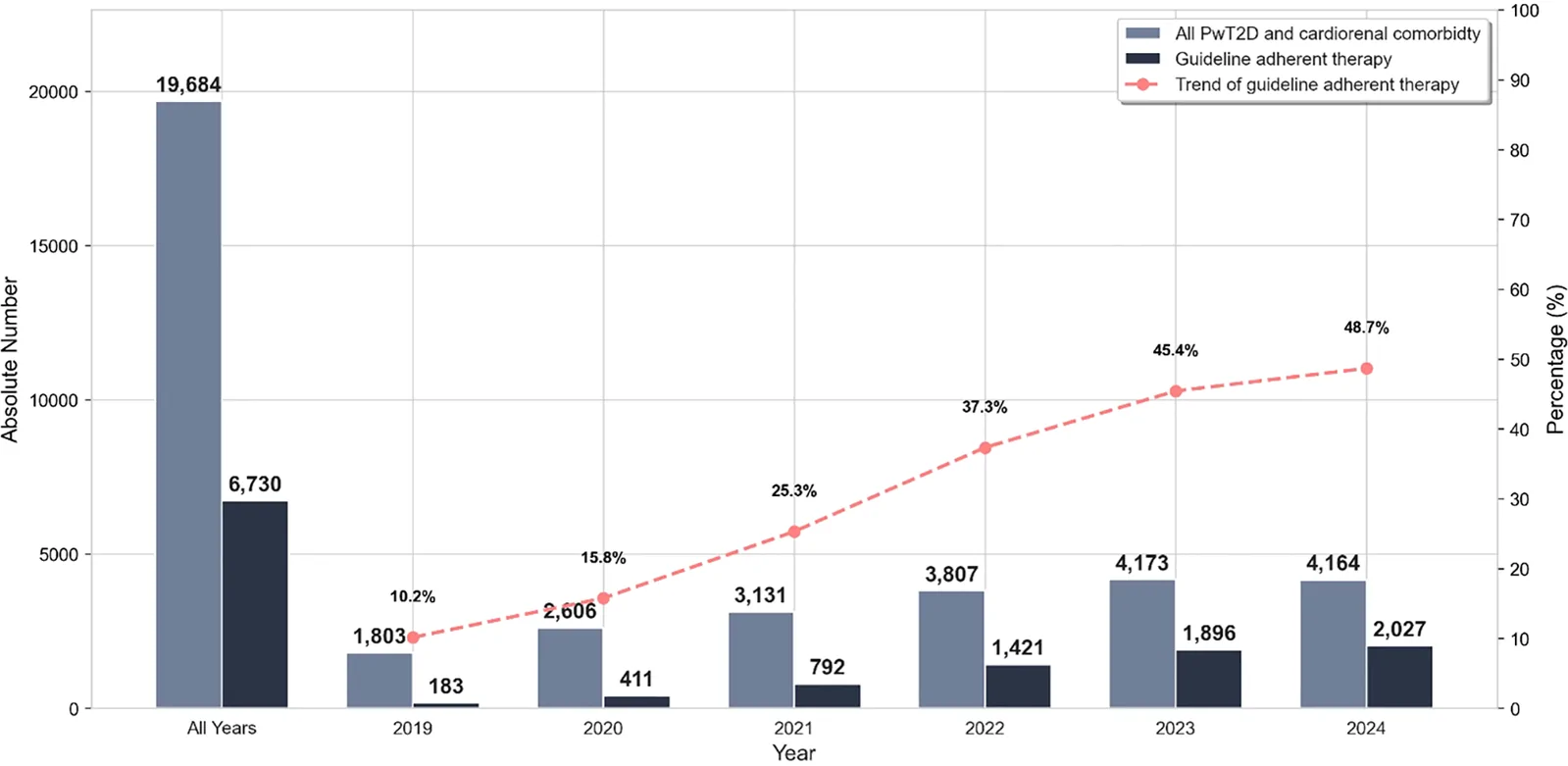
Temporal trends of guideline adherent therapy in PwT2D and cardiorenal comorbidity (N = 19,684). The number of all patients (grey bars) and of those receiving guideline adherent therapy (black bars) with corresponding proportion (red dashed line) are illustrated for all years and for each study year from 2019 to 2024 separately. X²-test showed significant linear increase in 2019-2024 (p<0.0001).

**Figure 3:**
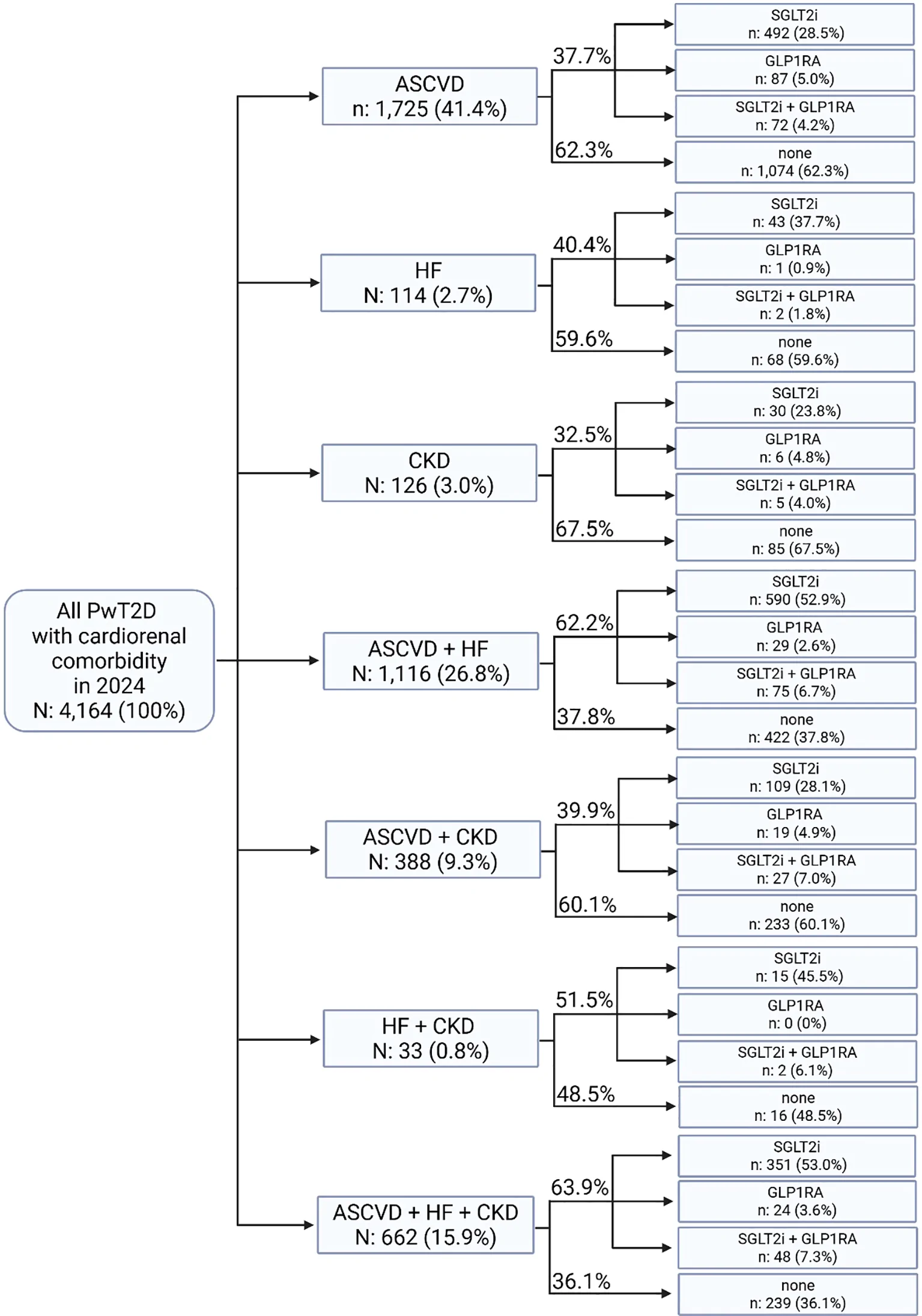
Use of SGLT2i and GLP1RA in PwT2D and cardiorenal comorbidity stratified by type of comorbidity (Cohort of 2024, n=4,164). SGLT2i, Sodium-glucose cotransporter-2 inhibitors; GLP1RA, glucagon-like peptide 1 receptor agonists; ASCVD, atherosclerotic cardiovascular disease; HF, heart failure; CKD, chronic kidney disease). Created in https://BioRender.com.

Annual medication rates were evaluated for the use of SGLT2i and/or GLP1RA for the total cohort of PwT2D and cardiorenal comorbidity (**Figure 2**, **Supplementary Table 1**). Trend rates were significantly rising during the observation period (p<0.0001) with an increase from 10.2% in 2019 to 48.7% in 2024. The greatest relative increase occurred in 2021, with a 1.6-fold rise, while the largest absolute increase was seen in 2022, at +12.0%. The smallest changes were observed between 2023 and 2024, with an absolute increase of only +3.3% and a corresponding relative increase of 1.07-fold. Among the identified cases of 4,164 PwT2D and cardiorenal comorbidity in the cohort of 2024, one comorbidity was documented in 41.4% (ASCVD/high risk for ASCVD or HF or CKD), two comorbidities in 36.9% and all three comorbidities in 15.8% of patients (ASCVD/high ASCVD risk and HF and CKD). By 2024, nearly every second PwT2D and cardiorenal comorbidity received a guideline adherent medication (2,027/4,164; 48.7%) with lowest rate in PwT2D and CKD (32.5%) and highest rates in PwT2D and ASCVD/high ASCVD risk and HF and CKD (63.9%). From a drug class perspective, the highest user rate for SGLT2i was found in those PwT2D with all three cardiorenal comorbidities (60.3%) and PwT2D with ASCVD/high ASCVD risk and HF (59.6%) and lowest in PwT2D and CKD (27.8%) and GLP1RA use was low in PwT2D and HF (2.6%) and ranging between 6.1% in PwT2D and HF and CKD to 11.9% in PwT2D and ASCVD/high ASVD risk and CKD. Combination therapy with SLGT2i and GLP1RA was found in a minority of patients (231/4164, 11.4%) with the highest rate in PwT2D and all three cardiorenal comorbidities (7.3%). (**Figure 3**)

### 3.3 Factors associated with guideline adherent therapy

**Figure 4:**
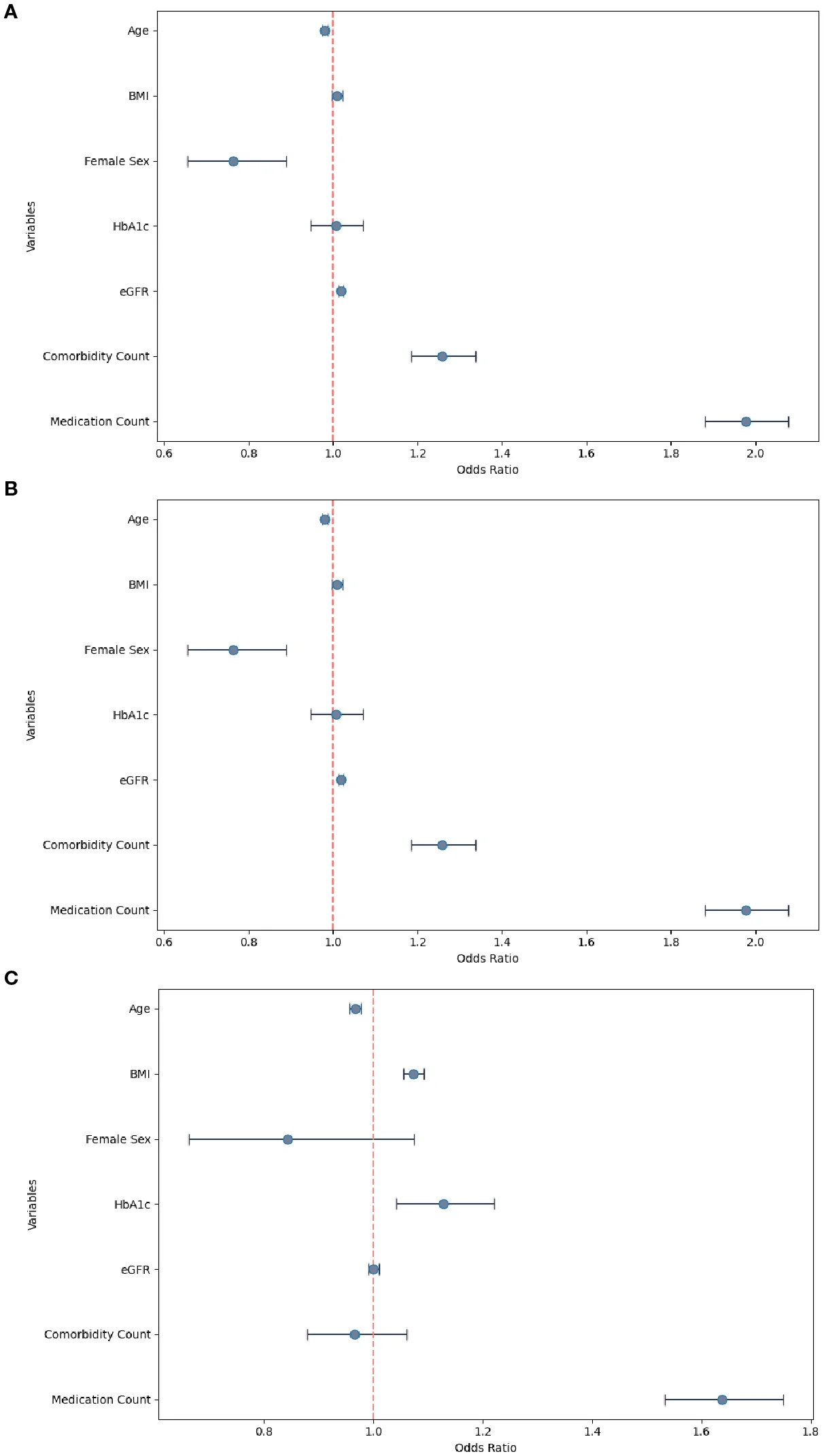
Factors associated with guideline adherent treatment in all PwT2D and cardiorenal comorbidity in 2024. Forest plots with adjusted ORs (dots), 95% confidence intervals (horizontal bars) and red dashed line presenting OR = 1.0. OR > 1.0 indicate higher chance and OR <1.0 indicate lower chance for use of **(A)** SGLT2i and/or GLP1RA, **(B)** SGLT2i and **(C)** GLP1RA. BMI, body mass index; HbA1c, glycosylated hemoglobin; eGFR, estimated glomerular filtration rate.

Several clinical factors were significantly linked with the chance for guideline adherent treatment (**Figure 4**). Analyzing the total cohort of PwT2D and cardiorenal comorbidity in 2024, women less likely to receive guideline adherent therapy (OR 0.76, 95% CI 0.66-0.89, p<0.001). Age was also linked to a lower chance for any organ-protective therapy (OR 0.98, 95% CI 0.99-0.97, p<0.0001). Number of comorbidities (OR 1.26, 95% CI 1.18-1.34, p<0.0001) and number of medication (OR 1.98, 95% CI 1.88-2.08, p<0.0001) were associated with higher chance for SGLT2i and/or GLP1RA treatment. High eGFR was associated with a slightly increased chance for guideline adherent treatment (OR 1.02, 95% CI 1.01-1.03, p<0.0001). (**Figure 3A**, **Supplementary Table 2A**)

Further analysis revealed drug class specific differences. For SGLT2i, female sex (OR 0.80, 95% CI 0.68-0.93, p=0.003) and to lesser extent age (OR 0.99, 95% CI 0.98-0.99, p<0.001) and BMI (OR 0.99, 95% CI 0.98-0.99, p=0.05) were associated with lower probability of being treated with SGLT2i. Number of medication (OR 1.02, 95% CI 1.83-2.01, p<0.0001), number of comorbidity (OR 1.29, 95% CI 1.22-1.37, p<0.0001) and with marginally effect also high eGFR (OR 1.02, 95% CI 1.01-1.03, p<0.0001) correlated with higher chance for SGLT2i therapy. (**Figure 3B**, **Supplementary Table 2B**) For GLP1RA, age was linked with slightly lower chance for GLP1RA treatment (OR 0.97, 95% CI 0.96-0.98, p<0.0001), while BMI (OR 1.07, 95% CI 1.05-1.09, p<0.0001), HbA1c (OR 1.13, 95% CI 1.04-1.22, p<0.01) and number of medication (OR 1.64, 95% CI 1.53-1.75, p<0.0001) were linked with increased chance for GLP1RA treatment. (**Figure 3C**, **Supplementary Table 2C**)

## 4 Discussion

Our study of nearly 20,000 PwT2D with cardiorenal comorbidities shows that, despite rising use of SGLT2i and GLP-1RA over time, guideline adherent therapy remains suboptimal, with substantial demographic and clinical differences - particularly by sex - and several factors such as age, comorbidity burden, and renal function significantly influencing treatment likelihood.

A Swedish Health registry and a Northeastern US health network analysis indicate that, following 2019 ADA/EASD consensus resp. 2019 ESC guidelines 50-80% of PwT2D are eligible for SGLT2i and/or GLP1RA therapy (6, 8). The observed increase in the medication rate of guideline adherent, organ-protective therapy during the observation period are consistent with a prior US American real-world analysis showing rising use of SGLT2i and GLP1RA in PwT2D populations at high risk for cardiorenal events with up to doubling of medication rates from 2018 through 2021 (9). Our study not only confirms this rising trend but also adds depth by spanning 2019–2024 and addressing a non-US cohort underlining that evidence-based promotion of SGLT2i and GLP1RA as first-line agents in PwT2D and high cardiorenal risk by guideline recommendations have translated into improved clinical care by time also in Germany. The observation of a more pronounced increase of medication rates during 2021–2022 in general and especially in those with HF are timely associated with publication of international guidelines recommending SGLT2i in PwT2D and CKD and in individuals with HF with reduced ejection fraction with and without diabetes (11–13).

Despite this encouraging trend, ten years after the first published cardiovascular outcome trial demonstrating clear cardiovascular benefit (14), a substantial treatment gap still persists. In contrast to U.S. studies and one Swedish study reporting that only 25% to 30% of eligible patients received guideline-directed therapy (6–9), we observed a higher rate of adherence. By 2024, approximately half of high-risk PwT2D in our cohort were treated in accordance with guideline recommendations. The greater uptake of organ-protective therapies in our study may, at least in part, reflect the management of patients within an academic tertiary-care setting, where specialist expertise and structured care pathways may facilitate earlier therapy initiation and optimization. However, the remaining treatment gap highlights ongoing barriers to implementation, even in settings with strong specialist involvement. Potential contributors include therapeutic inertia, competing clinical priorities, fragmented care between primary and specialty services, and limited patient awareness or concerns regarding newer drug classes (15).

Beyond these systemic challenges, our findings reveal a further layer of inequity: sex-based disparities in treatment were evident, with female patients being 33% less likely than men to receive evidence-based, organ-protective therapies with SGLT2i and/or GLP1RA. This observation aligns with prior work demonstrating sex-related differences in cardiometabolic risk profiles and care delivery, especially with lower odds for SGLT2i prescription (8, 16, 17). These sex differences may be attributable by agent specific risk profiles such as SGLT2i-associated risk for urinary tract infection which risk is more likely in women but may also reflect complex factors, including physician bias in cardiovascular risk perception, differing side-effect profiles, and healthcare access barriers. Such disparities emphasize the need for targeted implementation strategies to ensure equitable translation of evidence-based therapy across sexes. Lim and colleagues, in a large cross-sectional analysis from the JADE program, showed that women with T2D tend to present with a more adverse cardiometabolic risk profile and were less likely to achieve key treatment targets compared with men, partly attributable to differences in health behaviors and clinical management. These findings suggest that disparities in risk factor control and therapeutic intensity begin upstream of medication selection and may reflect both biological and sociocultural determinants of care (18).

Our finding that higher comorbidity burden and greater medication use were positively associated with therapy uptake aligns with the idea that patients with more complex disease are often prioritized for guideline-based treatment. This finding is line with Nanna et al. who described SGLT2i user as more often taking secondary preventive medication (9). Of note, in comparison to our results with comorbidity count being associated with higher prevalence of SGLT2i and GLP1RA intake, in this US cohort, individuals taking SGLT2i or GLP1RA were less frequently hospitalized in the previous year. These discrepancies may be explained by different care settings examined. While Nanna et al. included different levels of health care settings, we analyzed data from a tertiary care university hospital, thus our results may be explained by management by academic specialists (e.g., cardiologists or nephrologists) who might be more familiar with critical ill patients, recent guideline updates and evidence from major trials, which demonstrated clear cardiorenal benefits of SGLT2 inhibition. It is also possible that patients with multiple comorbidities have more frequent contact with healthcare systems, facilitating therapy optimization (19). However, this pattern also highlights a concerning inverse care gradient: patients with fewer comorbidities - who could benefit from early, preventive therapy - may remain undertreated. Our observations including GLP1RA-specific aspects with HbA1c and BMI linked to higher odds for GLP1RA use has been echoed in a Swedish Register Study finding that GLP1RA use was more likely in PwT2D and HF in presence of poor glucose control and long diabetes duration (20).

The strengths of the present study include the use of a large, interoperable FHIR-based dataset, allowing comprehensive capture of diagnoses (ICD-10 codes) and procedures (OPS codes), pharmacotherapy (ATC codes), and laboratory data over a five-year period. These features enabled robust analysis of temporal trends in administration patterns and the identification of patient-level factors associated with guideline adherent therapy. Limitations include the single-center design, which may restrict generalizability, and the observational single-defined time point rather than a prospective follow up nature of the study, which precludes causal inference. Additionally, administration records may not fully reflect actual medication adherence, and the dataset does not capture the clinical rationale for non-administration, including contraindications, patient preference, or prior adverse events or year of diabetes diagnosis.

Taken together, our results show that clinical practice is responsive to emerging evidence and guideline recommendations, but also reinforce that inequities in the allocation of organ-protective therapies persist, even when guideline indications are clear. While clinicians increasingly recognize the value of these therapies in patients with advanced disease, significant implementation gaps remain in the general population of PwT2D and cardiorenal disease but especially among women with T2D. Understanding whether these disparities stem from clinician prescribing patterns, sex-specific differences in perceived risk, patient preferences, or systemic biases within care pathways will be essential for designing targeted interventions. Overcoming these barriers may require structured, system-level strategies, including decision-support tools, audit-and-feedback mechanisms, and enhanced awareness by education including special aspects such as a sex-specific health care gap, to ensure equitable cardiometabolic protection for all eligible patients.

## 5 Conclusion

Guideline adherent therapy reflecting the paradigm shift from a glucocentric approach to organ-protective treatment concepts is improving in Germany, yet a substantial care gap remains, leaving every second patient inadequately treated. Future research should investigate the causality and impact of clinical factors to better tailor strategies that support the implementation of guideline recommendations in routine care and thereby enhance overall quality of care.

## Data Availability

The data analyzed in this study is subject to the following licenses/restrictions: Patient data are not publicly available due to privacy concerns and legal requirements. Data supporting the conclusions of this article will be made available by the authors upon reasonable request. Requests to access these datasets should be directed to Jens.Kleesiek@uk-essen.de.
