# Correction: Implementation of guideline-recommended organ-protective therapy in people with type 2 diabetes and cardiovascular disease, heart failure, or kidney disease: real-world evidence from a German University Hospital

**DOI:** 10.3389/fendo.2026.1954561

**Published:** 2026-10-05

**Authors:** Karzan Suliman, Young Hee Lee-Barkey, Alexander Brehmer, Bernd Stratmann, Jasmin M. Klose, Tim Lenfers, Jens Kleesiek, Susanne Reger-Tan, Julius Keyl

**Affiliations:** 1 Department of Diabetes and Endocrinology, Heart and Diabetes Center NRW, University Hospital of Ruhr-University Bochum, Bochum, Germany; 2 Institute for Artificial Intelligence in Medicine (IKIM), University Hospital Essen, Essen, Germany; 3 Institute of Pathology, University Hospital Essen, Essen, Germany

**Keywords:** atherosclerotic cardiovascular disease, chronic kidney disease, GLP1RA, guidelines, heart failure, organ protection, SGLT2i, type 2 diabetes

The equal contribution mark (†) was erroneously applied to all authors; it should apply only to “Karzan Suliman and Young Hee Lee-Barkey”.

The original version of this article has been updated.

