# Supplementary material for: Implementation of guideline-recommended organ-protective therapy in people with type 2 diabetes and cardiovascular disease, heart failure, or kidney disease: real-world evidence from a German University Hospital
Source: Front Endocrinol (Lausanne). 2026 Jan 6;16:1736984. doi: 10.3389/fendo.2025.1736984 (PMC12816769; doi:10.3389/fendo.2025.1736984)
Supplement: Supplementary file 1 [file DataSheet1.docx]

Supplementary Material

# Supplementary Data

**1.1 Cohort Extraction**

| **Base Cohort** | |
| --- | --- |
| All patients with ICD E11 * from 2019-2024 |  |
|  | |
| **Electronic Health Record Value Codes** | |
| 39156-5 | BMI (if not calculated from height + weight) |
| 29463-7 | height |
| 8302-2 | weight |
| zHBA1C | hba1c |
| zTRIG | triglyceride |
| zCHOL | cholesterol |
| zHDL | hdl_cholesterol |
| zLDL | ldl_cholesterol |
| zGOT | ASAT |
| zGPT | ALAT |
| zKREA | creatinine Jaffe |
| zGFR | GFR |
| 85354-9 | 'bp' (systolic + diastolic) |
|  | |
| **Conditions** | |
| E11 | type 2 diabetes |
| I25 | coronary artery disease |
| I20 | angina pectoris |
| I21 | acute myocardial infarction |
| I63 | stroke |
| I70 | atherosclerosis |
| E66 | obesity (OR BMI >=30) |
| I10 | hypertension |
| I15 | secondary hypertension |
| I50 | heart failure |
| N18 | chronic kidney disease |
| E78 | dyslipidemia |
| R80 | albuminuria |
| ASCVD = True if:   coronary artery disease \| myocardial infarction \| stroke \| atherosclerosis \| coronary revascularization \| transluminal angioplasty   OR (AGE >=55 AND 2 risk factors (obesity, hypertension, smoking, dyslipidemia, albuminuria)) | |
|  | |
| **Procedures** | |
| 5-363.4 | Operations on the coronary vessels: other revascularization of the heart: revascularization with free internal mammary artery graft (IMA graft) |
| 8-981 | Other complex multimodal treatment: complex neurological treatment of acute stroke |
| 8-836 | Therapeutic intravascular catheterization and cannulation: (Percutaneous) transluminal angioplasty |
| 8-837 | Therapeutic intravascular catheterization and cannulation: Percutaneous transluminal angioplasty of heart and coronary arteries |
| 8-849 | (Percutaneous) transluminal stent implantation: (Percutaneous) transluminal implantation of other large-bore bare-metal stents |
|  | |
| **Medication** | |
| atc_a10a_codes = [  # A10AB - Fast-acting insulins and analogues  "A10AB01", "A10AB02", "A10AB03", "A10AB04", "A10AB05", "A10AB06", "A10AB30",  # A10AC - Intermediate-acting insulins and analogues  "A10AC01", "A10AC02", "A10AC03", "A10AC04",  # A10AD - Combinations of intermediate- and fast-acting insulins  "A10AD01", "A10AD02", "A10AD03", "A10AD04", "A10AD05", "A10AD06", "A10AD30",  # A10AE - Long-acting insulins and analogues  "A10AE01", "A10AE02", "A10AE03", "A10AE04", "A10AE05", "A10AE06",  "A10AE07", "A10AE30", "A10AE54", "A10AE56",  # A10AF - Insulins and analogues for inhalation  "A10AF01",  ]  atc_a10b_codes = [  # A10BA - Biguanides  "A10BA01", "A10BA02", "A10BA03",  # A10BB - Sulfonylureas  "A10BB01", "A10BB02", "A10BB03", "A10BB04", "A10BB05", "A10BB06",  "A10BB07", "A10BB08", "A10BB09", "A10BB10", "A10BB11", "A10BB12",  "A10BB31",  # A10BC - Sulfonamides (heterocyclic)  "A10BC01",  # A10BD - Combinations of oral blood glucose lowering drugs  "A10BD01", "A10BD02", "A10BD03", "A10BD04", "A10BD05", "A10BD06",  "A10BD07", "A10BD08", "A10BD09", "A10BD10", "A10BD11", "A10BD12",  "A10BD13", "A10BD14", "A10BD15", "A10BD16", "A10BD17", "A10BD18",  "A10BD19", "A10BD20", "A10BD21", "A10BD22", "A10BD23", "A10BD24",  "A10BD25", "A10BD26", "A10BD27", "A10BD28", "A10BD29", "A10BD30",  # A10BF - Alpha-glucosidase inhibitors  "A10BF01", "A10BF02", "A10BF03",  # A10BG - Thiazolidinediones  "A10BG01", "A10BG02", "A10BG03", "A10BG04",  # A10BH - DPP-4 inhibitors  "A10BH01", "A10BH02", "A10BH03", "A10BH04", "A10BH05", "A10BH06",  "A10BH07", "A10BH08", "A10BH51", "A10BH52",  # A10BJ - GLP-1 analogues  "A10BJ01", "A10BJ02", "A10BJ03", "A10BJ04", "A10BJ05", "A10BJ06",  "A10BJ07",  # A10BK - SGLT2 inhibitors  "A10BK01", "A10BK02", "A10BK03", "A10BK04", "A10BK05", "A10BK06",  "A10BK07", "A10BK08", "A10BK09", "A10BK10",  # A10BX - Other blood glucose lowering drugs  "A10BX01", "A10BX02", "A10BX03", "A10BX05", "A10BX06", "A10BX08",  "A10BX15", "A10BX16", "A10BX17", "A10BX18"  ]  atc_c10_codes = [  # C10AA - HMG CoA reductase inhibitors (statins)  "C10AA01", "C10AA02", "C10AA03", "C10AA04", "C10AA05", "C10AA06",  "C10AA07", "C10AA08",  # C10AB - Fibrates  "C10AB01", "C10AB02", "C10AB03", "C10AB04", "C10AB05", "C10AB06",  "C10AB07", "C10AB08", "C10AB09", "C10AB10", "C10AB11", "C10AB12",  # C10AC - Bile acid sequestrants  "C10AC01", "C10AC02", "C10AC03", "C10AC04",  # C10AD - Nicotinic acid and derivatives  "C10AD01", "C10AD02", "C10AD03", "C10AD04", "C10AD05", "C10AD06",  "C10AD52",  # C10AX - Other lipid modifying agents  "C10AX01", "C10AX02", "C10AX03", "C10AX05", "C10AX06", "C10AX07",  "C10AX08", "C10AX09", "C10AX10", "C10AX11", "C10AX12", "C10AX13",  "C10AX14", "C10AX15", "C10AX16", "C10AX17", "C10AX18", "C10AX19",  # C10BA - Combinations (statins + other lipid agents)  "C10BA01", "C10BA02", "C10BA03", "C10BA04", "C10BA05", "C10BA06",  "C10BA07", "C10BA08", "C10BA09", "C10BA10",  # C10BX - Combinations (statins + other drugs)  "C10BX01", "C10BX02", "C10BX03", "C10BX04", "C10BX05", "C10BX06",  "C10BX07", "C10BX08", "C10BX09", "C10BX10", "C10BX11", "C10BX12",  "C10BX13", "C10BX14", "C10BX15", "C10BX16", "C10BX17", "C10BX18",  "C10BX19", "C10BX20", "C10BX21"  ]  atc_c02_codes = [  # C02A - Antiadrenergic agents, centrally acting  "C02AA01", "C02AA02", "C02AA03", "C02AA04", "C02AA05", "C02AA06", "C02AA07",  "C02AA52", "C02AA53", "C02AA57", "C02AB01", "C02AB02", "C02AC01", "C02AC02",  "C02AC04", "C02AC05", "C02AC06",  # C02C - Antiadrenergic agents, peripherally acting  "C02CA01", "C02CA02", "C02CA03", "C02CA04", "C02CA06", "C02CC01", "C02CC02",  "C02CC03", "C02CC04", "C02CC05", "C02CC06", "C02CC07",  # C02D - Arteriolar smooth muscle, agents acting on  "C02DA01", "C02DB01", "C02DB02", "C02DB03", "C02DB04", "C02DC01", "C02DD01",  "C02DG01",  # C02K - Other antihypertensives  "C02KA01", "C02KB01", "C02KD01", "C02KD02", "C02KD03", "C02KD04", "C02KD05",  "C02KX01", "C02KX02", "C02KX03", "C02KX04", "C02KX05", "C02KX06",  # C02L - Antihypertensives and diuretics in combination  "C02LA01", "C02LA02", "C02LA03", "C02LA04", "C02LA50", "C02LA51", "C02LA71",  "C02LB01", "C02LC01", "C02LC05", "C02LC51", "C02LE01", "C02LF01", "C02LG01",  "C02LG51", "C02LG73", "C02LK01",  # C02N - Combinations of antihypertensives in ATC-group C02  "C02NA01",  ]  c03_codes = ['C03', 'C03A', 'C03B', 'C03C', 'C03D', 'C03E', 'C03X', 'C03AA', 'C03AA01', 'C03AA02', 'C03AA03', 'C03AA04', 'C03AA05', 'C03AA06', 'C03AA07', 'C03AA08', 'C03AA09', 'C03AA13', 'C03AA56', 'C03AB', 'C03AB01', 'C03AB02', 'C03AB03', 'C03AB04', 'C03AB05', 'C03AB06', 'C03AB07', 'C03AB08', 'C03AH', 'C03AH01', 'C03AH02', 'C03AX', 'C03AX01', 'C03B', 'C03BA', 'C03BA02', 'C03BA03', 'C03BA04', 'C03BA05', 'C03BA07', 'C03BA08', 'C03BA09', 'C03BA10', 'C03BA11', 'C03BA12', 'C03BA13', 'C03BA82', 'C03BB', 'C03BB02', 'C03BB03', 'C03BB04', 'C03BB05', 'C03BB07', 'C03BC', 'C03BC01', 'C03BD', 'C03BD01', 'C03BK', 'C03BX', 'C03BX03', 'C03C', 'C03CA', 'C03CA01', 'C03CA02', 'C03CA03', 'C03CA04', 'C03CB', 'C03CB01', 'C03CB02', 'C03CC', 'C03CC01', 'C03CC02', 'C03CD', 'C03CD01', 'C03CX', 'C03CX01', 'C03D', 'C03DA', 'C03DA01', 'C03DA02', 'C03DA03', 'C03DA04', 'C03DA05', 'C03DB', 'C03DB01', 'C03E', 'C03EA', 'C03EA01', 'C03EA02', 'C03EA03', 'C03EA04', 'C03EA05', 'C03EA06', 'C03EA07', 'C03EB', 'C03EB01', 'C03EB02', 'C03EB03', 'C03X', 'C03XA', 'C03XA01']  c07_codes = ['C07', 'C07A', 'C07B', 'C07C', 'C07D', 'C07F', 'C07A', 'C07AA', 'C07AA01', 'C07AA02', 'C07AA03', 'C07AA05', 'C07AA06', 'C07AA07', 'C07AA09', 'C07AA12', 'C07AA14', 'C07AB', 'C07AB02', 'C07AB03', 'C07AB04', 'C07AB05', 'C07AB06', 'C07AB07', 'C07AB08', 'C07AB09', 'C07AB10', 'C07AB11', 'C07AB12', 'C07AB13', 'C07AB14', 'C07AB15', 'C07AC', 'C07AC01', 'C07AC02', 'C07AC03', 'C07AC04', 'C07AC05', 'C07AC06', 'C07AG', 'C07AG01', 'C07AG02', 'C07AG03', 'C07B', 'C07BA', 'C07BA01', 'C07BA02', 'C07BA03', 'C07BA04', 'C07BA05', 'C07BA06', 'C07BA07', 'C07BA08', 'C07BB', 'C07BB02', 'C07BB03', 'C07BB04', 'C07BB05', 'C07BB06', 'C07BB07', 'C07BB09', 'C07BB10', 'C07BB12', 'C07BB52', 'C07C',  'C07CA', 'C07CA01', 'C07CA02', 'C07CA03', 'C07CA04', 'C07CB', 'C07CB01', 'C07CB02', 'C07CB03', 'C07CB04', 'C07CB05', 'C07CB06', 'C07CB07', 'C07D', 'C07DA', 'C07DA06',  'C07DB', 'C07DB01', 'C07FB', 'C07FB02', 'C07FB03', 'C07FB04', 'C07FB05', 'C07FB06', 'C07FB07', 'C07FB08', 'C07FB09', 'C07FB10', 'C07FB11', 'C07FX', 'C07FX01', 'C07GA', 'C07GA01', 'C07GA02', 'C07GA03']  c08_codes = ['C08', 'C08A', 'C08B', 'C08C', 'C08D', 'C08E', 'C08G', 'C08A', 'C08AA', 'C08AA01', 'C08AA02', 'C08AA03', 'C08AA04', 'C08AA05', 'C08AA06', 'C08AA07', 'C08AA08', 'C08AA09', 'C08AA10', 'C08AA12', 'C08AA13', 'C08AA14', 'C08AA15', 'C08AA16', 'C08AA17', 'C08AA18', 'C08AA51', 'C08AA52', 'C08AA53', 'C08AA54', 'C08AA55', 'C08AA56', 'C08AA57', 'C08AA58', 'C08AA59', 'C08AA66', 'C08AA67', 'C08AB', 'C08AB01', 'C08AB02', 'C08AB03', 'C08AB04', 'C08AB05', 'C08AB06', 'C08AB07', 'C08AB08', 'C08AB09', 'C08AB10', 'C08AB11', 'C08AB12', 'C08AB13', 'C08AB14', 'C08AB15', 'C08AB16', 'C08AB17', 'C08AB18', 'C08AB19', 'C08AB20', 'C08AB21', 'C08AB51', 'C08AB52', 'C08AB53', 'C08AB54', 'C08AB55', 'C08AB56', 'C08AB57', 'C08AB58', 'C08AB59', 'C08AB60', 'C08AB61', 'C08AB63', 'C08AB71', 'C08B', 'C08BA', 'C08BA01', 'C08BA02', 'C08BA03', 'C08BA04', 'C08BA05', 'C08BA06', 'C08C', 'C08CA', 'C08CA01', 'C08CA02', 'C08CA03', 'C08CA04', 'C08CA05', 'C08CA06', 'C08CA07', 'C08CA08', 'C08CA09', 'C08CA10', 'C08D', 'C08DA', 'C08DA01', 'C08DA02', 'C08DA03', 'C08DA04', 'C08DA05', 'C08DA06', 'C08DA07', 'C08E', 'C08EAA', 'C08EAA01', 'C08EAA02', 'C08EAA03', 'C08EAA04', 'C08EAB', 'C08EAB01', 'C08EAB02', 'C08G', 'C08GA', 'C08GA01', 'C08GA02']  c09_codes = ['C09', 'C09A', 'C09B', 'C09C', 'C09D', 'C09X', 'C09A', 'C09AA', 'C09AA01', 'C09AA02', 'C09AA03', 'C09AA04', 'C09AA05', 'C09AA06', 'C09AA07', 'C09AA08', 'C09AA09', 'C09AA10', 'C09AA11', 'C09AA12', 'C09AA13', 'C09AA14', 'C09AA15', 'C09B', 'C09BA', 'C09BA01', 'C09BA02', 'C09BA03', 'C09BA04', 'C09BA05', 'C09BA06', 'C09BA07', 'C09BA08', 'C09BA09', 'C09BA10', 'C09BA11', 'C09BA12', 'C09BA13', 'C09BA14', 'C09BA15', 'C09BB', 'C09BB02', 'C09BB03', 'C09BB04', 'C09BB05', 'C09BB06', 'C09BB07', 'C09BB08', 'C09BB09', 'C09BB10', 'C09BB12', 'C09BB13', 'C09BB14', 'C09BX', 'C09BX01', 'C09BX02', 'C09BX03', 'C09BX04', 'C09BX05', 'C09BX06', 'C09BX07', 'C09BX08', 'C09BX09', 'C09BX10', 'C09BX11', 'C09C', 'C09CA', 'C09CA01', 'C09CA02', 'C09CA03', 'C09CA04', 'C09CA05', 'C09CA06', 'C09CA07', 'C09CA08', 'C09CA09', 'C09CA10', 'C09CA11', 'C09CA12', 'C09CA13', 'C09CA14', 'C09CA15', 'C09CA16', 'C09D', 'C09DA', 'C09DA01', 'C09DA02', 'C09DA03', 'C09DA04', 'C09DA05', 'C09DA06', 'C09DA07', 'C09DA08', 'C09DA09', 'C09DA10', 'C09DA11', 'C09DB', 'C09DB01', 'C09DB02', 'C09DB04', 'C09DB05', 'C09DB06', 'C09DB07', 'C09DB08', 'C09DB09', 'C09DX', 'C09DX01', 'C09DX02', 'C09DX03', 'C09DX04', 'C09DX05', 'C09DX06', 'C09DX07', 'C09DX08', 'C09DX09', 'C09DX10', 'C09DX11', 'C09DX12', 'C09X', 'C09XA', 'C09XA02', 'C09XA03', 'C09XA53'] | |

# Supplementary Figures and Tables

|  | 2019 | 2020 | 2021 | 2022 | 2023 | 2024 | p-value for linearity over time |
| --- | --- | --- | --- | --- | --- | --- | --- |
| PwT2D and cardiorenal comorbidity (n=19,684) | | | | | | | |
| GLP1RA and/or  SGLT2i | 183 | 411 | 792 | 1,421 | 1,896 | 2,027 | <0.0001 |
| (%) | 10.15 | 15.77 | 25.3 | 37.33 | 45.43 | 48.68 |  |

**Supplementary Table 1 -** Prescription rates of SGLT2i and/or GLP1RA in PwT2D with cardiorenal comorbidity 2019-2024. Analysis for trend over time by Х^2^-test for trend.

A SGLT2i and/or GLP1RA

| Parameter | Odds Ratio (OR) | OR lower CI | OR upper CI | p-value |
| --- | --- | --- | --- | --- |
| Age | 0.98 | 0.97 | 0.99 | <0.0001 |
| BMI | 1.01 | 1.00 | 1.02 | 0.15 |
| Female Sex | 0.76 | 0.66 | 0.89 | <0.001 |
| HbA1c | 1.01 | 0.95 | 1.07 | 0.83 |
| eGFR | 1.02 | 1.01 | 1.03 | <0.0001 |
| Comorbidity Count | 1.26 | 1.18 | 1.34 | <0.0001 |
| Medication Count | 1.98 | 1.88 | 2.08 | <0.0001 |

B SGLT2i

| Parameter | Odds Ratio (OR) | OR lower CI | OR upper CI | p-value |
| --- | --- | --- | --- | --- |
| Age | 0.99 | 0,98 | 0.99 | \| <0.001 \| \| --- \| |
| BMI | 0.99 | 0.98 | 1.00 | 0.05 |
| Female Sex | 0.80 | 0.68 | 0.93 | 0.003 |
| HbA1c | 0.99 | 0.93 | 1.06 | 0.81 |
| eGFR | 1.02 | 1.01 | 1.03 | <0.0001 |
| Comorbidity Count | 1.29 | 1.22 | 1.37 | <0.0001 |
| Medication Count | 1.92 | 1.83 | 2.01 | <0.0001 |

C GLP1RA

| Parameter | Odds Ratio (OR) | OR lower CI | OR upper CI | p-value |
| --- | --- | --- | --- | --- |
| Age | 0.97 | 0.96 | 0.98 | <0.0001 |
| BMI | 1.07 | 1.05 | 1.09 | <0.0001 |
| Female Sex | 0.84 | 0.66 | 1.07 | 0.17 |
| HbA1c | 1.13 | 1.04 | 1.22 | <0.01 |
| eGFR | 1.00 | 0.99 | 1.01 | 0.99 |
| Comorbidity Count | 0.97 | 0.88 | 1.06 | 0.46 |
| Medication Count | 1.64 | 1.53 | 1.75 | <0.0001 |

**Supplementary Table 2 -** Multivariate logistic regression analyses showing associations between patient characteristics and use of guideline-adherent treatment in PwT2D and cardiorenal comorbidity 2024 for (A) SGLT2i and/or GLP1RA, (B) SGLT2i, (C) GLP1RA. Odds ratios (OR) with 95% confidence intervals (CI).
